# Modular polymer platform as a novel approach to head and neck cancer therapy

**DOI:** 10.1038/s41598-022-07324-y

**Published:** 2022-03-04

**Authors:** Yazeed Alhiyari, Jundong Shao, Albert Y. Han, Amanda Miller, Jeffrey F. Krane, Marie Luff, Milica Momcilovic, David Shackelford, Zhen Gu, Maie A. St. John

**Affiliations:** 1 Department of Head and Neck Surgery, David Geffen School of Medicine, University of California, 10833 Le Conte Ave, 62-132 CHS, Los Angeles, CA 9009 USA; 2 Department of Bioengineering, University of California Los Angeles, Los Angeles, CS USA; 3 Jonsson Comprehensive Cancer Center, University of Los Angeles Medical Center, Los Angeles, CA USA; 4 David Geffen School of Medicine, University of Los Angeles, Los Angeles, CA USA; 5 DGSOM Division of Pulmonary and Critical Care Medicine, Head and Neck Cancer Program, University of Los Angeles, Los Angeles, CA USA; 6 Department of Pathology and Laboratory Medicine, UCLA Medical Center, Los Angeles, CA USA

**Keywords:** Cancer, Cancer therapy, Head and neck cancer

## Abstract

Head and neck cancer is the sixth most common cancer in the world, with more than 300,000 deaths attributed to the disease annually. Aggressive surgical resection often with adjuvant chemoradiation is the cornerstone of treatment. However, the necessary chemoradiation treatment can result in collateral damage to adjacent vital structures causing a profound impact on quality of life. Here, we present a novel polymer of poly(lactic-co-glycolic) acid and polyvinyl alcohol that can serve as a versatile multidrug delivery platform as well as for detection on cross-sectional imaging while functioning as a fiduciary marker for postoperative radiotherapy and radiotherapeutic dosing. In a mouse xenograft model, the dual-layered polymer composed of calcium carbonate/thymoquinone was used for both polymer localization and narrow-field infusion of a natural therapeutic compound. A similar approach can be applied in the treatment of head and neck cancer patients, where immunotherapy and traditional chemotherapy can be delivered simultaneously with independent release kinetics.

## Introduction

### Clinical justification

Head and neck cancer is the sixth most common cancer in the world, with more than 70,000 cases diagnosed in the United States ever year^1^. During the past 30 years, the three-to five-year survival rate of patients with advanced/locally recurrent head and neck squamous cell carcinoma (HNSCC) has remained poor (20–30%)^2^ despite considerable advances in surgical techniques, irradiation delivery, chemotherapeutic strategies, as well as these treatments used in combination. Approximately 50% of patients do not survive their disease and fail primary management; thus, salvage of the recurrent cancer patient remains of paramount importance^3^. Although palliative chemotherapy is often attempted, systemic toxicity and its impact on the quality of life prevents its wider clinical application^4^. Treatments that allow for the reduced dose delivery and exposure to healthy tissue while providing equivalent tumor control compared to conventional therapies would vastly improve the patient’s survival and the survivorship process.

### Use of multilayered polymer for drug delivery

Delivering a lethal dose of radiation to a tumor while sparing nearby normal tissues remains a great challenge in radiation therapy. Interstitial brachytherapy, which involves the surgical implantation of radiation sources (afterload catheters or seeds) in and around a tumor, followed by the delivery of continuous low-dose radiation at a limited treatment volume, have been used^5–7^. The radiation seeds placed at the time of the operation, such as ^125^I or ^103^Pd, expose personnel to radiation and proper protection must be used^8^. Such treatments can also require the patient to be isolated while the therapy is ongoing. Additionally, the catheters or seeds can move from their original positions, thus affecting the radiation dose administered to the tumor bed and the normal surrounding tissues^9^. Some advantages of an implanted polymer system include better control of the dose distribution, the elimination of radioprotection and safety issues for the patient as well as the patient’s family and the treating personnel. An important psychological factor is that the patient’s daily activities are not restricted during the entire treatment time. An additional benefit of this polymer system includes prophylaxis against tumor recurrence following resection. Viable squamous cell carcinoma cells have previously been recovered from the surgical wound following neck dissection and have been shown to be capable of growing as colonies in vitro; theoretically, these may implant and cause cancer recurrence^10^.

The combined use of radiotherapy and chemotherapy has been effective in improving the therapeutic index of radiation therapy for a variety of human cancers^11,12^. Previous studies have reported that the intratumoral administration of chemotherapeutic agents such as cisplatin, in a sustained-release drug delivery system markedly improved antitumor efficacy and reduced systemic toxicity compared with systemically administered drugs^13^. Indeed, even in our previous work, we demonstrated that a poly(e-caprolactone):poly(lactide-cocaprolactone) polymer film loaded with cisplatin can be used for delivering targeted local chemotherapy in a partially resected xenograft animal model of head and neck cancer^14^.

### Choice of calcium carbonate and TQ

In this study, we used the combinatorial approach of a dual-layer polymer, employing CaCO_3_ for the imaging-based facilitation of radiotherapy (RT) and thymoquinone (TQ), a radiosensitizing agent, to enhance RT. Calcium carbonate is an inert material that is easily detected on cross-sectional imaging. In addition, it is cheaper and industrially feasible compared with other high-Z metals, such as gold nanoparticles. While operating, surgeons can easily determine in which anatomic areas a tumor was adherent vs. being easily removed. Intraoperatively, the surgeon can place the polymer in the areas of closely adherent tumor, thus guiding the radiation therapist’s fields more precisely. Patients can then receive precision treatment, through image guided localization of the polymer implant, minimizing side effects of radiation therapy. Thymoquinone (TQ), a natural product derived from black seed oil, is shown to have anti-cancer properties while being biologically well-tolerated^15–18^. We established that in a partially resected xenograft animal model of head and neck squamous cell carcinoma the dual-layer CaCO_3_/TQ polymer platform demonstrated a significant reduction in the tumor volume and offered an avenue for the precise localization of areas in the wound bed for adjunct RT.

## Results

### Cells vs TQ and radiation

**Figure 1:**
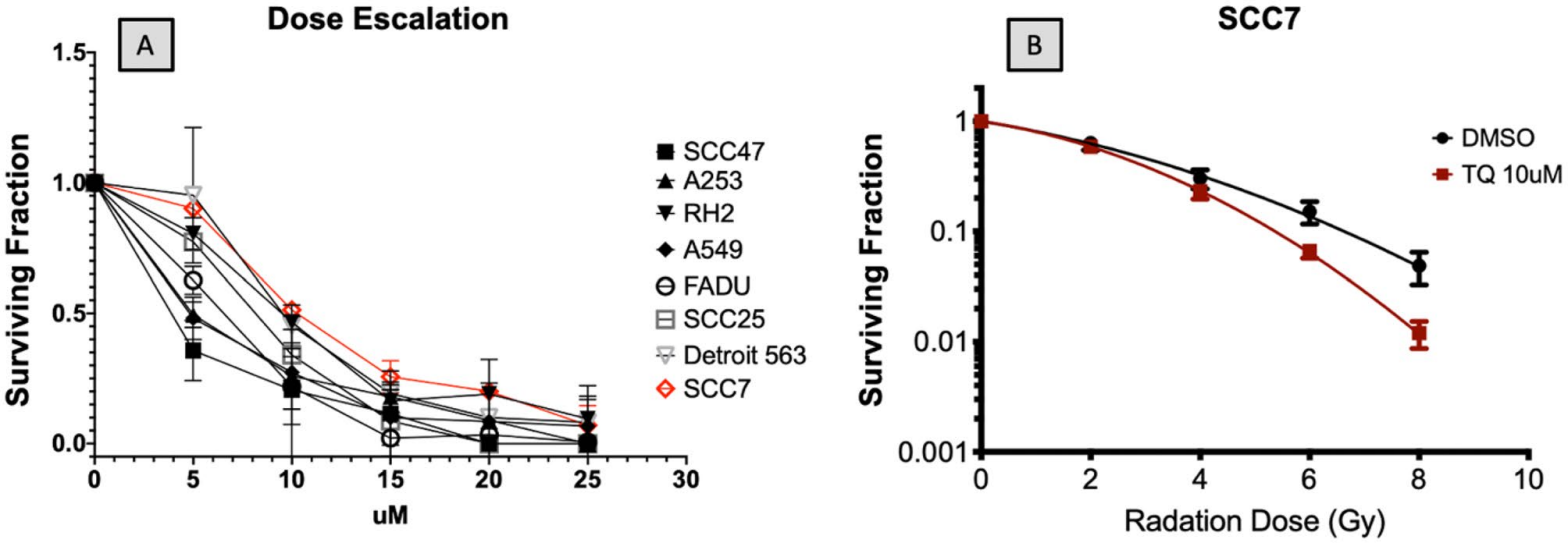
TQ treatment decreases cancer cell survival. (**A**) Dose escalation studies using a panel of human head and neck cell lines and SCCVIISF mouse derived line, demonstrates that 15 μM of TQ is sufficient for reducing colony formation by 50% or more (*P* < 0.001). (**B**) Linear quadratic survival curve depicting TQ-treated SCCVIISF vs control. TQ synergizes with radiation causing a significant decrease in cancer cell survival at 8 Gy with *p* < 0.05 and with 4.86% (SD 2.7%) for RT only and 1.2% (SD 0.6%) for RT in combination with 10 μM TQ.

For determining the optimal TQ dose required to induce LD50, a dose escalation experiment was performed using a panel of one mouse and six human head and neck cell lines: SCCVIISF, FADU, SCC47, A253, Detroit 563, SCC25 and, two squamous cell lung cancer cell lines RH2, A549 respectively. Across all cell lines, a 15 μmol dose of TQ achieved a statistically significant (*p* < 0.001) LD50 or better as observed via a cell colony formation (clonogenic assay) (Fig. 1A). Therefore, we aimed to achieve a dose of at least 15 μM as the effective dose in the drug delivery polymer layer for the in vitro cell assay. In order to calculate the total dosage, polymers were weighed before implantation (after surgical debulking) and again 30 days later at the termination of the experiment (after removal). The change in the weight reflected the absolute diffusion of TQ from the polymer film. Through this, it was estimated that the total dose delivered over a period of 30 days was 23 mg with an average dose delivery rate of 0.77 mg per day or 4.68 μmol/uM^3^. The mouse cell line was treated with 10 μmol of TQ and irradiated at 2, 4, 6 and 8 Gy to demonstrate that TQ plus radiation had a greater cell killing effect over radiation alone (Fig. 1B). The surviving fraction at 8 Gy was significant with *p* < 0.05 and with 4.86% (SD 2.7%) for RT only, and 1.2% (SD 0.6%) for RT in combination with 10 μmol of TQ.

### Polymer characteristics

**Figure 2:**
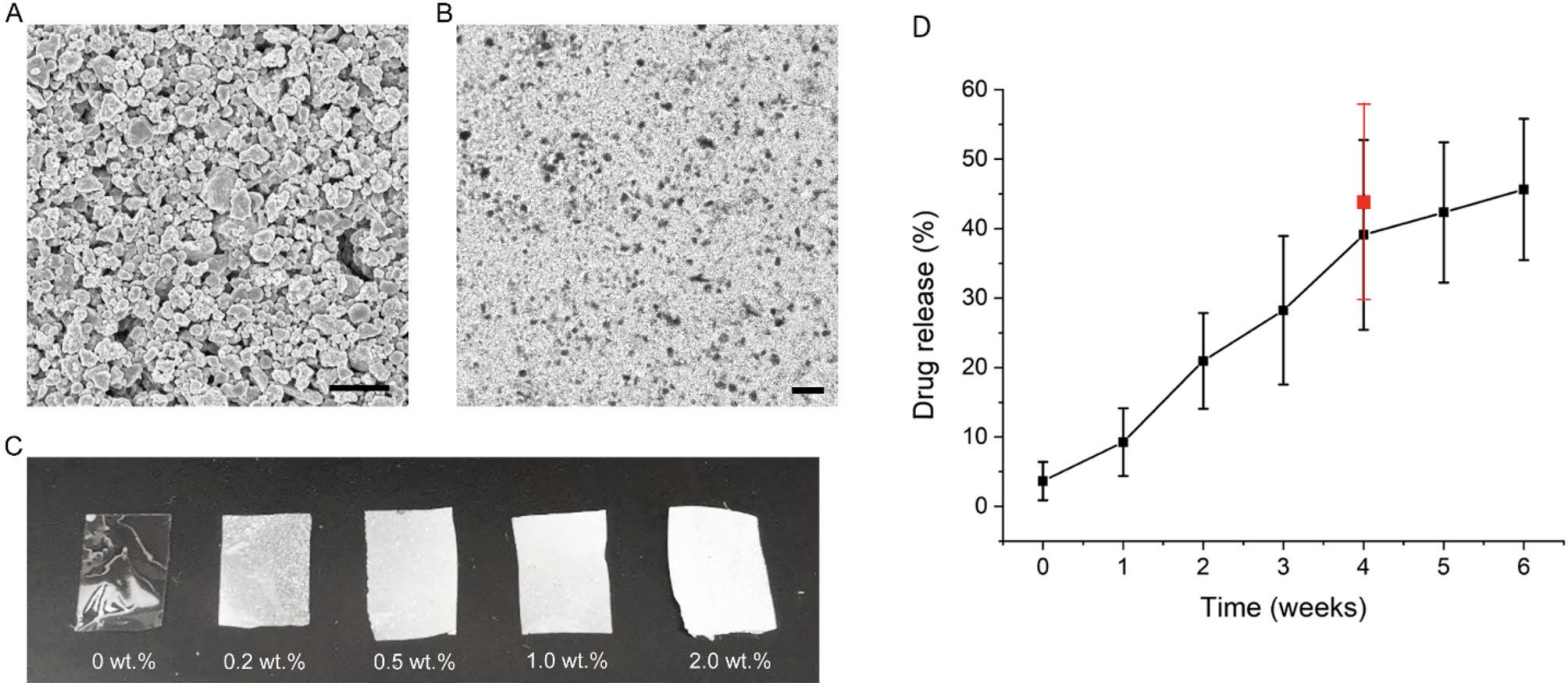
Characterization of CaCO_3_ micron particles and CaCO_3_/PVA films and release kinetics. (**A**) SEM image of CaCO_3_ micron particles (scale bar: 50 μm). (**B**) Microscopic image of CaCO_3_/PVA film (scale bar: 50 μm). (**C**) CaCO_3_/PVA films with varying concentrations of CaCO_3_ (0, 0.2, 0.5, 1.0, and 2.0 wt%). (**D**) Drug release characteristics measured in vitro (black line) while red point represents drug release upon collection from mouse terminal surgery.

**Figure 3:**
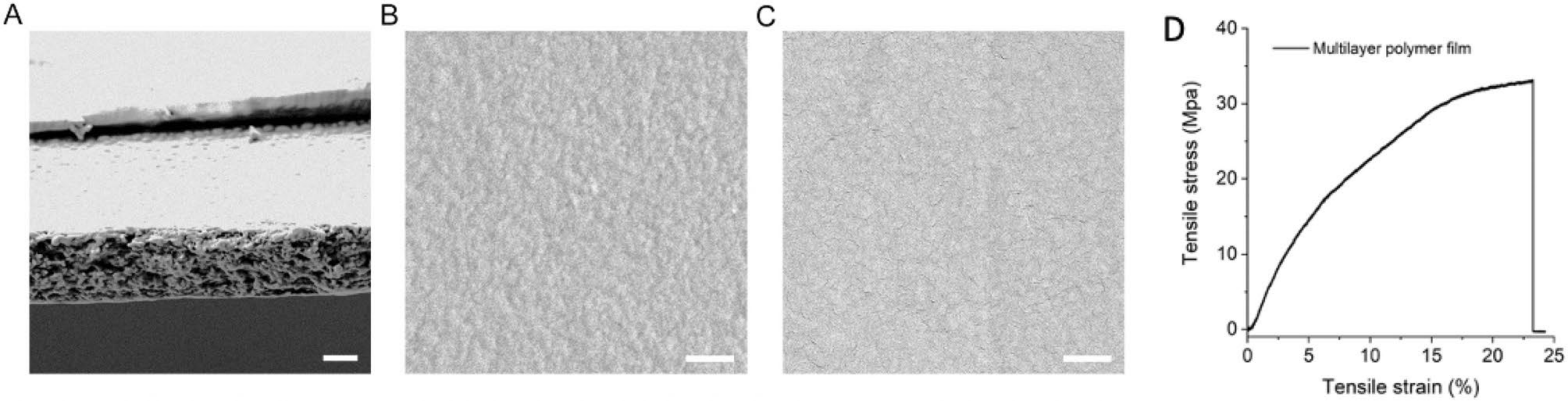
Characterizations of multilayer polymer film. (**A**) SEM image of the cross-section of multilayer polymer film (scale bar: 50 μm). (**B**) and (**C**) SEM images of the surface of CaCO_3_/PVA layer and TQ/PLGA layer from multilayer polymer film (scale bar: 5 μm). (Dual-layer polymer blend with treatment layer loaded with TQ and imaging layer composed of CaCO_3_). In (**D**) mechanical performance of the multiplayer polymer film is depicted as tensile strain vs tensile stress. The tensile strength of the multilayer polymer film was measured using dynamic *mechanical* analysis; the film has a very high tensile strength (32.9 Mpa), which is useful for clinical applications.

**Figure 4:**
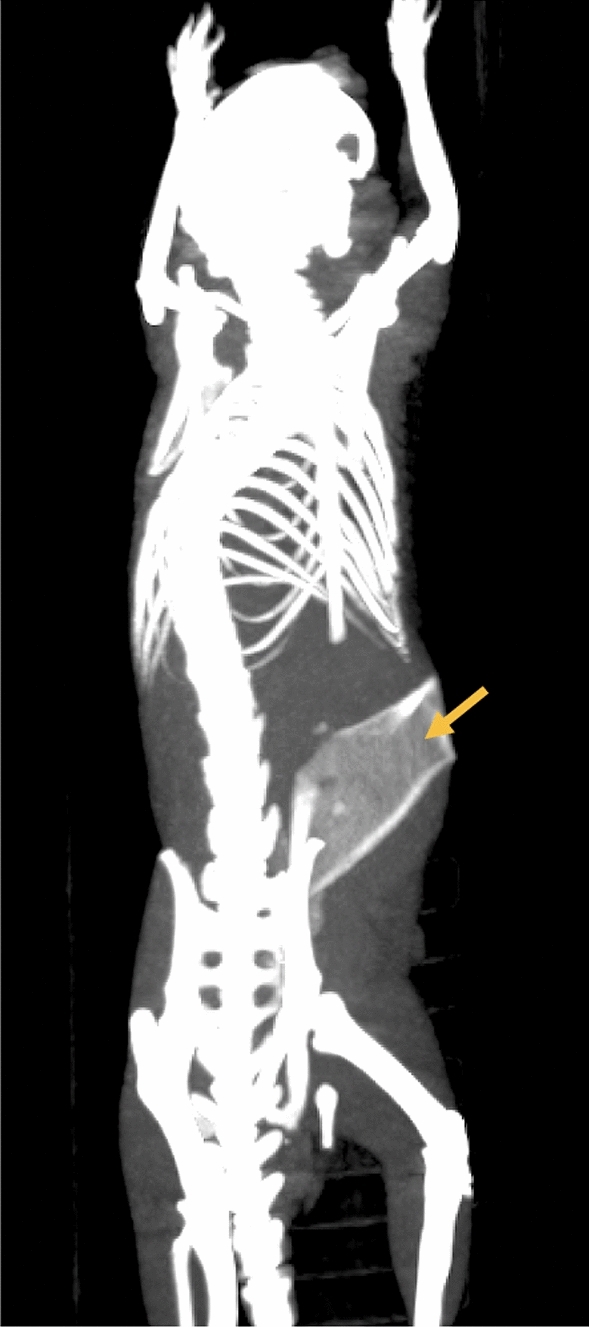
CT imaging demonstrating polymer localization in the tumor bed for precision radiotherapy localization.

The assessment of the polymer film using scanning electron microscopy (Figs. 2 and 3) (SEM) image of CaCO_3_ micron particles in Fig. 2A reveals a uniform morphology with an average particle size of approximately 20 μm. As shown in Fig. 2B, C, in-situ CaCO_3_ micron particles were evenly distributed in the polyvinyl alcohol (PVA) film. The SEM image in Fig. 3A shows the cross-section topography of the multilayer polymer film. The film shows a two-layer structure with a lateral size of 120 μm and 30 μm for the CaCO_3_/PVA layer and TQ/PLGA layer, respectively. SEM images of the CaCO_3_/PVA layer and TQ/PLGA layer with a smooth surface morphology are shown in Fig. 3B, C. Figure 4 demonstrates that the concentration of CaCO_3_ can easily be visualized using computed tomography (CT) scan with an equal effect at 1.0 wt%.

### Visual examination

**Figure 5:**
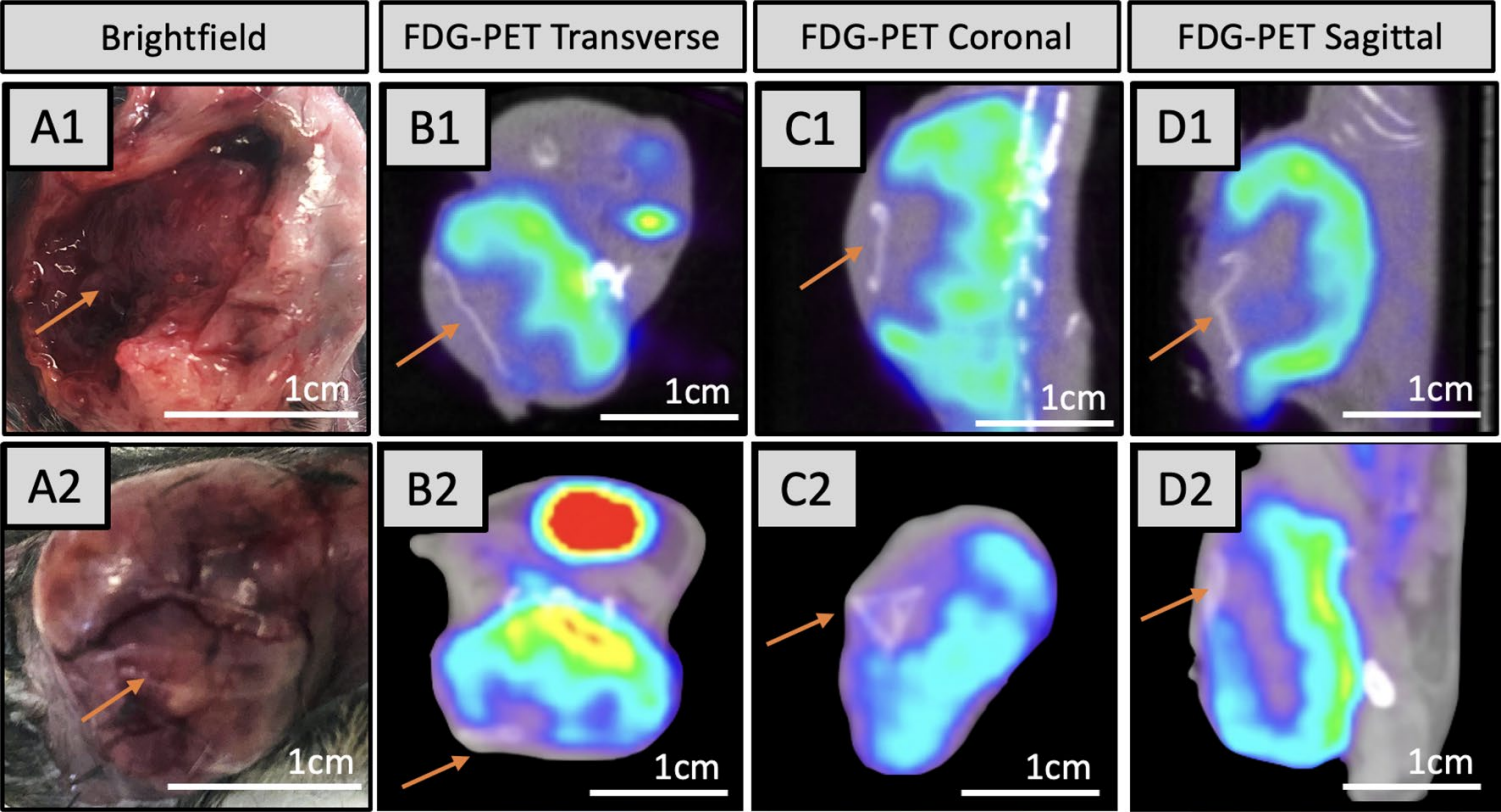
Localization of polymer via Gross and FDG-PET imaging. (**A1**) Tumor exposed with TQ polymer removed. (**B1**–**D1**) demonstrate transverse, coronal, and sagittal sections of tumor in (**A1**). FDG-PET signal is decreased around the TQ-embedded polymer treated areas with no tumor regrowth. Arrows mark location of polymer. The polymer imaging layer is visible in a standard FDG-PET scan. (**A2**) Depicts exposed tumor upon which plain control polymer (lacking TQ drug layer) was placed. (**B2**–**D2**) are the corresponding PET images of (**A2**). (**A1**) mouse tumor had 0.024%ID/g FDG-PET uptake, whereas (**A2)** has 0.055%ID/g.

**Figure 6:**
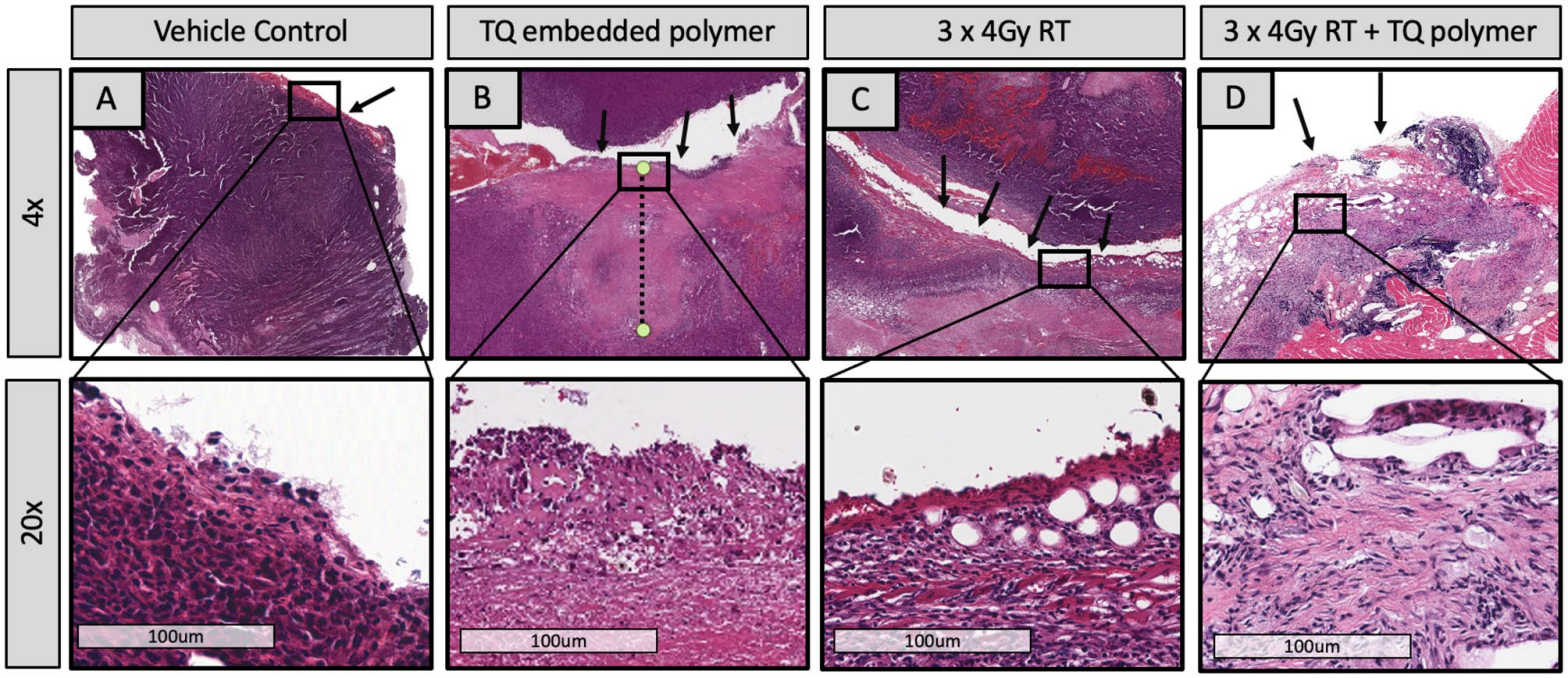
Histological assessment of the tumor at the site of polymer removal. (**A**–**D**) demonstrates tumors collected from four different mouse treatment groups: vehicle control, TQ-embedded polymer, 3 × 4 Gy RT, and 3 × 4 Gy + TQ polymer from left to right with their corresponding insets below. Arrows signify the location of the polymer upon tumor removal. (**A**) vehicle control: Poorly differentiated carcinoma with viable tumor persists. Along the polymer boundary, a thin layer of dark pink fibrotic and inflammatory tissue can be observed. (**B**) TQ-embedded polymer: Tumor necrosis is seen with no viable tumor in the treatment area; The pink areas correspond to necrosis seen extending below the area of the drug treatment layer (dotted line). (**C**) 3 × 4 Gy RT : Narrow band of necrosis is noted along the polymer site with viable pockets of tumor tissue below. (**D**) 3 × 4 Gy + TQ polymer: No viable tumor present.

At the termination of the experiment (30 days post-polymer implantation) a necropsy was performed for both the treatment polymer group and the control polymer group, and tissues were examined grossly, radiographically and histologically (Figs. 5 and 6). Vehicle control tumors (polymers with no drugs and no RT) had persistence of poorly differentiated carcinomas with completely viable tumors when evaluated microscopically at 40 × with H&E staining. Poorly differentiated cancers appear very abnormal and lack the organizational structures of the tissues they reside in. (Fig. 6). TQ polymer-treated groups demonstrated tumor killing with tumor necrosis along the drug treatment polymer layer (Fig. 6).

### Pathological examination

In order to study pathological changes between the treatment groups, histology was performed to evaluate differences at the cellular level. The control group, which received the polymer without TQ or radiotherapy, showed dense nuclei at the polymer placement site, revealing persistent poorly differentiated carcinoma with viable tumor (Fig. 6A). The group treated with the TQ polymer only (Fig. 6B) revealed no tumor regrowth with tumor necrosis and no viable tumor in the treatment area. In contrast, the radiotherapy-only group presented with a narrow band of necrosis along the polymer removal site with still-viable portions of tumor tissue found below (Fig. 6C). The TQ polymer with radiotherapy group (Fig. 6D) presented with no residual tumor present.

### FDG-PET/CT assessment

FDG-PET/CT imaging was used to assess tumor metabolic activity and to identify the polymer location relative to the tumor. Figure 5B1–D1 demonstrate FDG-PET/CT imaging with decreased uptake of the PET tracer observed in areas of the tumor directly in contact with the drug-delivering polymer edge. This was in contrast with the control polymer group, which did not receive TQ drug treatment (Fig. 5B2–D2); where robust cancer persists. TQ-polymer-treated uptake of FDG-PET was half that of the vehicle control with 0.024%ID/g vs 0.055%ID/g, respectively.

### Tumor growth dynamics

**Figure 7:**
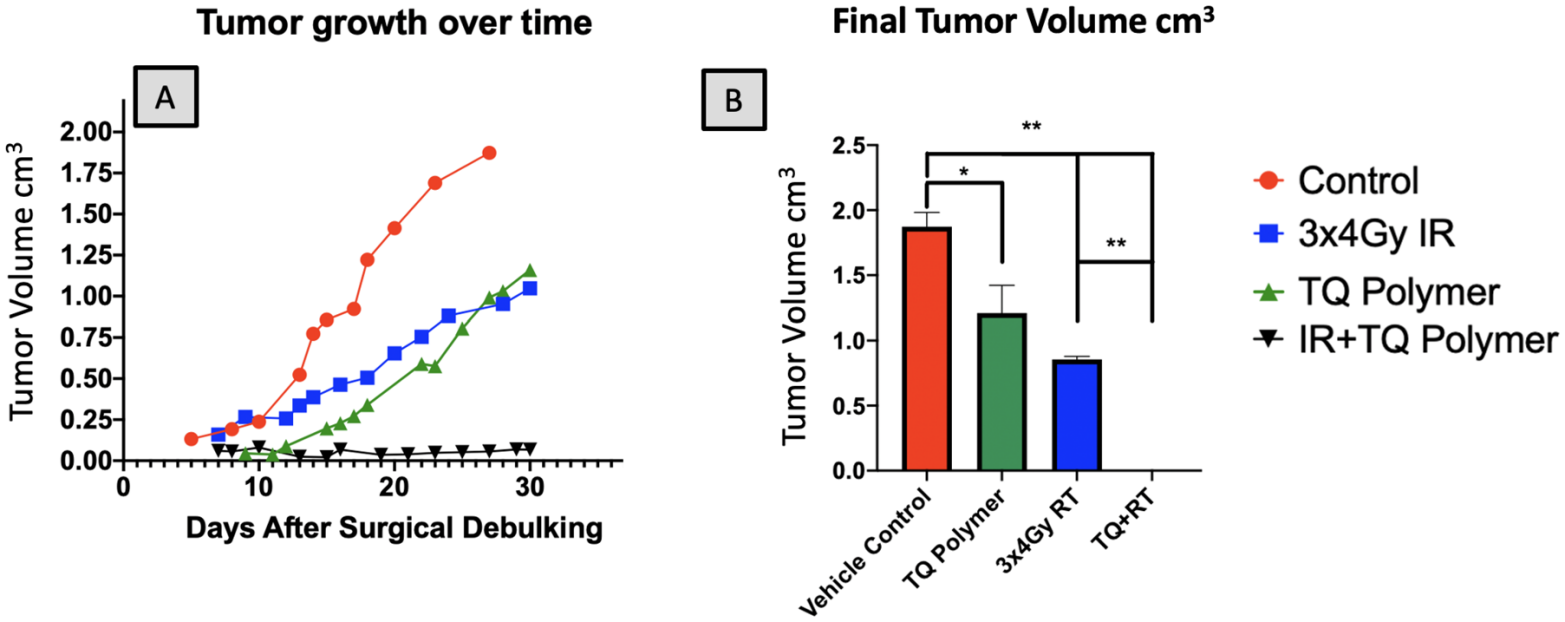
Final volume measurements. Tumor volume growth after 50% surgical debulking per each treatment group. *N* >  = 4; validated three times. (**A**) Shows tumor measurements taken by calipers over the course of 30 days post-surgical debulking. (**B**) Tumor volume upon resection. *p < 0.05, **p < 0.001, error bars are plotted as SEM. TQ + fractionated RT reflects no physically measurable tumor by caliper assessment; this was confirmed by histological analysis, where no residual cancer was seen (Fig. 6).

In order to assess the overall changes in tumor volume, each tumor was measured across their length, width, and height using calipers. In an analysis of the growth rate over the course of 30 days, the radiotherapy plus TQ polymer groups revealed the slowest rate of change (Fig. 7A), and upon terminal histological examination no viable tumor was found. In review with our expert Head and Neck pathology team, this minimal change was likely due to caliper measurement variations due to inflammation and wound healing post treatment. A statistically significant difference was found in the tumor volume when the control polymer group was compared with all other treatment groups (Fig. 7B, *P* = 0.002). The radiotherapy plus TQ polymer groups demonstrated the smallest tumor volumes overall. RT vs RT + TQ polymer treatment proved significant with *p* < 0.05.

## Discussion

### Significance of this study

In this study, we demonstrated that a modular, dual-layer CaCO_3_/TQ polymer provided effective anti-cancer treatment in in vivo cancer xenograft models. A multilayer polymer allowed for the individual loading and adjustment of drugs for cancer treatment. The polymer film physically adheres to the tumor bed contours when placed surgically, thus allowing the drug delivery layer to come into direct contact with the remaining cancer tissues and or cancer bed. The interaction of the polymer film and the tumor surface, along with the slow biodegradation of the polymer promoting localized drug delivery throughout the RT therapy, demonstrated a significant decrease in the tumor growth rate when compared with other groups over the course of the experiment.

### Novelty of using TQ in polymer

Our choice of loading TQ in the radiosensitizing drug layer was substantiated by its previously demonstrated anticancer efficacy^15,16^. TQ is a biologically well-tolerated compound^15^, which was further exhibited in our mice, as they showed no local tissue injury at the site of implantation. Our data also demonstrates that the polymer’s ability to provide the sustained release of TQ is what drives the therapeutic benefit observed in our study. TQ has been shown to inhibit cancer cell proliferation; arrest cancer cell cycle progression; and, induce pro-apoptotic effects with the targets of Bcl-2, caspases, PPArs, NF-kB, STAT3, MAPK, Akt and ROS^16^. Polymers containing therapeutic agents that can be released slowly over time would continue to provide therapeutic benefit even for microscopic disease (clinical R1 resection) unable to be detected by clinical imaging or visualization. The ability to manipulate the duration of drug release, and the order of drugs released from multiple layers can add great clinical utility in the treatment of patients.

The combinational approach of RT and the local release of chemotherapy has been shown to be an effective therapeutic strategy, especially as the local administration of chemotherapeutics does not contribute to systemic side effects^19^. Our platform makes use of this principle, allowing for the synergistic optimization of RT via local radiosensitizing drug delivery to the cancer cells left behind following surgical debulking. The polymer platform allows for a significant reduction in tumor volume and offers an avenue for the precise localization of areas in the wound bed for adjunct RT.

The use of multiple layers composed of various properties (PVA and PLGA) provides the ability to modulate the chemotherapeutic drug delivery rate and the lifetime of the imaging layer independently. The approach is similar to poly (N-isopropylacrylamide) (PNIPAAm) and PLGA for separate release mechanisms^20^. The modular platform allows for multiple layers that deliver controlled and targeted release of radiosensitizers, targeted inhibitors, and immunomodulators. Previous studies demonstrated that magnetic-based core–shell particles with the sustained release of curcumin and the temperature-dependent release of doxorubicin was effective in a melanoma orthotopic mouse model^21^. With the modification of the fabrication process of PVA and PLGA polymers, the controlled release may last as long as seven months^22^.

### Use of CaCO_3_ for polymer localization

One novel feature of the study design was the use of CaCO_3_ for visualization on cross-sectional imaging. In prior studies, inert metals or radiopaque hydrogel (e.g., TraceIT Tissue Marker) have been used as fiducial markers for image-guided radiotherapy, external beam radiation and brachytherapy localization^23,24^. The benefit of using CaCO_3_ is the use of a natural compound that is absorbed by the body over time^25^. This is the first study of its kind to demonstrate the utility of embedded CaCO_3_ in a polymer as a potential fiduciary marker for image-guided radiotherapy. While operating, surgeons can easily determine in which anatomic areas a tumor was adherent vs. being easily removed. Intraoperatively, the surgeon can place the polymer in the areas of closely adherent tumor, thus guiding the radiation therapists’ fields more precisely. The flexibility of the polymer sheet allows for it to deform with the tissues it resides. The polymers’ flexibility is a unique feature compared to traditional markers used for localization. The shape of the polymer sheet within the patient can then guide conformal radiation therapy minimizing secondary side effects of radiotherapy.

## Conclusion

The management of head and neck cancer patients poses a considerable challenge to the surgeon and the radiation oncologist. Herein, we report a novel dual-layer polymer film that is capable of delivering antitumor therapeutic agents with significant tumor kill. This polymer wrap was designed to be applied intraoperatively to the surgical bed after removing or debulking the tumor, thus allowing for enhanced post-operative radiation treatment, and also functioning as a platform for the delivery of therapeutic agents and immunomodulators. Besides its clinically relevant features, the modular nature of this polymer platform provides an elegant approach to future investigations, allowing us to seek out specific molecular targets. We have the capacity to build polymers that specifically target each patient’s tumor as we can profile the tumors and then target them selectively on the polymer platform. As more combinations of the polymer platform are developed, direct polymer therapy may play an important role in the armamentarium against oral and head and neck carcinoma, as well as many other cancers and human diseases.

## Methods

### Cell culture

Head and neck cancer cell line panels were ordered from ATCC and mouse derived squamous cell carcinoma, SCCVIISF, was spontaneously captured from a mouse tumor and grown in culture. All cell lines were cultured in the following conditions: 37 °C, 5% CO_2_ incubator with Dulbecco’s Modified Eagle’s Medium (DMEM), 10% fetal bovine serum (FBS), and 1% penicillin/streptomycin. The media was changed twice a week until the cells were confluent. Confluent cells were then passaged or harvested for use.

### Synthesis of multilayer films

The Poly(D,L-lactide-co-glycolide) (PLGA, 50:50, Mn 25,000), polyvinyl alcohol (PVA, MW: 31,000–50,000), and dichloromethane (DCM) were purchased from Sigma-Aldrich (Santa Barbara, USA). The phosphate buffered saline (pH 7.4) was obtained from Gibco Life Technologies (AG, Switzerland). All of the chemicals used in this study were analytic reagent grade and were used without further purification.

Multilayer films were prepared layer-by-layer, and the fabrication of each layer depended on various factors of the material, including its rigidity, toughness, adhesion, degradability, porosity, hydrophilicity, and loading capacity, among others. One fabrication technique involved a mechanical spinner followed by heat and evaporation of the solvent. First, 10 g of the PVA powder were added to 100 mL of deionized water and then centrifuged for 1 h at 95 °C to obtain a 10% (w/v) PVA solution. Then, 0.4 g of calcium carbonate (CaCO_3_) microparticles were dispersed in 10 mL of a PVA solution after bath sonication for 5 min and the mixture was vortexed for 2 min (Fig. 1). The obtained homogeneous solution was dropped onto a plastic petri dish and placed in a shaker so that the solution could be spread evenly. Subsequently, the petri dish was transferred to an oven at 70 °C for 2 h to ensure that the water was completely evaporated. Finally, the first layer (CaCO3/PVA film) for CT imaging was obtained. Furthermore, 0.5 g of the PLGA powders were added in 10 mL of DCM and then centrifuged for 30 min to obtain 5% (w/v) PLGA solution. Then, 20 mg of the TQ powders were dispersed in 2 mL of a PLGA solution after bath sonication for 5 min and the mixture was vortexed for 2 min. The resulting mixture was dropped on the first layer in the petri dish and then evaporated with ambient air under proper ventilation for 30 min. The multilayer polymer film was imaged using scanning electron microscopy (Fig. 2). Tensile stress was measured for the polymer as shown in Fig. 3. Finally, multilayer films with the first layer (CaCO_3_/PVA film) for CT imaging and the second layer (TQ/PLGA film) for chemotherapy were obtained (Fig. 4).

### Mouse surgery protocol

C3H/HeJ male mice (The Jackson Laboratories, Bar Harbor, ME, USA) were used in the study (Animal Research Committee (ARC), protocol number 2008-147). The Chancellor’s Animal Research Committee of the University of California, Los Angeles, and the Animal Research: Reporting In Vivo Experiments (ARRIVE)^26^ guidelines and protocols were approved and followed. For testing the polymer platform, 20 8-week-old C3H/HeJ mice were injected with 400,000 cells of a mouse derived squamous cell carcinoma line, SCCVIISF, in the right rear flank. Tumor growth was assessed with calipers three times per week following polymer implantation for 18–31 days to evaluate the antitumor efficacy of the different treatments. The control mice required euthanasia as determined by the Animal Research Committee of University of California at Los Angeles and in accordance with the American Veterinary Medical Association (AVMA) guidelines for the euthanasia of Animals (2020)^27^ due to the tumor burden. The lengths, widths, and heights (in mm) of the tumors were measured and the tumor volume (cm^3^) was calculated according to the formula: *Tumor volume* = *π/6* × *length* × *width* × *height*. When tumors reached an average size of 0.5–1 cm, all animals underwent surgery to debulk their tumors by 50%. This was done to approximate the surgical situation when a patient’s tumor is unresectable, and some tumor is left behind prior to polymer therapy. Animals were then randomly assigned to the various treatment groups. The treatment groups included: (1) Inert polymer with no drug; (2) TQ polymer; (3) Inert polymer + 3 × 4 Gy RT; (4) TQ polymer + 3 × 4 Gy RT. No systemic TQ treatment was given. Each tumor bed was covered with 2.25 cm^2^ of the polymer cut in the shape of the remaining tumor. The polymer was draped over the tumor edges and sutured in place.

### Radiation therapy

Mice in the RT group were anaesthetized with ketamine/xylazine and had eye ointment applied to their eyes for days 1, 2, and 3 post-surgery for RT treatment. The mice were positioned under ½ inch of lead shielding leaving only the tumor exposed. An X-ray dose was delivered at 0.4299 Gy/min for 9.3 min until 4 Gy of the total dose was received. Mice received 3 × 4 Gy RT, which is the scaled comparable dose given to head and neck cancer patients.

### MicroPET imaging

In vivo small animal imaging was conducted at the Crump Institute’s Preclinical Imaging Technology Center. The mice were injected via the lateral tail vein with a radiotracer (70 μCi for 18F-FDG and 200–400 μCi for 11C-L-glutamine), and they then underwent 60 min of uptake under 2% isoflurane anesthesia, followed by microPET (G8 PET/CT, PerkinElmer) and microCT (CrumpCAT, Arion Hadjioannou laboratory) imaging. The quantification of 18F-FDG uptake was done using AMIDE software by drawing a region of interest over the tumor and the entire body, as well as by calculating each of the maximum uptake values (SUVmax) as the percent of the injected dose per gram (%ID/g).

### Tissue collection

After the animals were sacrificed, a gross necropsy examination was conducted of the surrounding tissues. No discernable differences at the implant site could be observed between the groups. Tumor specimens were collected for sectioning and hemotoxin and eosin staining were done through the UCLA Translational Core Pathology Lab. Histopathological examination was performed with the assistance of a senior pathologist at UCLA Medical Center.

### Estimation of TQ dose rate delivery

Imbedded polymers were weighed before implantation and upon resection. The imaging and drug treatment layer were peeled apart and weighed separately, as the imaging layer was formulated to last three months where the drug treatment layer was formulated to last 1–1.5 months. Subtracting the difference in weights from before and after one month of implantation, we found that the CaCO_3_ imaging layer lost 5% of its weight and the drug treatment layer lost 60% of its weight, equating to an estimated delivery of 0.77 mg per day or 4.68 uM/uM^3^.

### Statistics

The statistical significance was set at *P* = 0.05. The dose-escalation experiment for the optimal TQ dose required to induce LD50 across eight cell lines was generated via a fitted, non-linear regression curve and determined via a one-sample t-test. The tumor volume was compared between the treatment groups with a one-way analysis of variance model. In addition, the statistical significance of differences in the tumor volume when comparing the control group with all other treatment groups was determined via an unpaired two-sample t-test. A linear quadratic survival curve depicting TQ-treated SCCVIISFs versus the control was analyzed via GraphPad Prism.
